# Supplementary material for: Resolving the full spectrum of human genome variation using Linked-Reads
Source: Genome Res. 2019 Apr;29(4):635–45. doi: 10.1101/gr.234443.118 (PMC6442396; doi:10.1101/gr.234443.118)
Supplement: Supplemental Material [file supp_gr.234443.118_Supplemental_longranger-2.2.zip › longranger-2.2.tar.bz2!/lr2.2-public/lib/stan/cmdstan/stan/doc/api/html/error__codes_8hpp_source.html]

Stan: src/stan/gm/error\_codes.hpp Source File


|  |  |
| --- | --- |
|  | Stan  2.5.0  probability, sampling & optimization |


- Main Page
- Related Pages
- Namespaces
- Classes
- Files

- File List
- File Members

All Classes Namespaces Files Functions Variables Typedefs Enumerations Enumerator Friends Macros Pages

- src
- stan
- gm

error\_codes.hpp

Go to the documentation of this file.

1 #ifndef STAN\_\_GM\_\_ERROR\_CODES\_HPP

2 #define STAN\_\_GM\_\_ERROR\_CODES\_HPP

3

4 namespace stan {

5  namespace gm {

6

7  struct error\_codes {

8  // defining error codes to follow FreeBSD sysexits conventions

9  // http://www.gsp.com/cgi-bin/man.cgi?section=3&topic=sysexits

10  enum {

11  OK = 0,

12  USAGE = 64,

13  DATAERR = 65,

14  NOINPUT = 66,

15  SOFTWARE = 70,

16  CONFIG = 78

17  };

18  };

19  }

20 }

21 #endif

stan::gm::error\_codes::SOFTWARE

**Definition:** error\_codes.hpp:15

stan::gm::error\_codes::NOINPUT

**Definition:** error\_codes.hpp:14

stan::gm::error\_codes::OK

**Definition:** error\_codes.hpp:11

stan::gm::error\_codes

**Definition:** error\_codes.hpp:7

stan::gm::error\_codes::USAGE

**Definition:** error\_codes.hpp:12

stan::gm::error\_codes::DATAERR

**Definition:** error\_codes.hpp:13

stan::gm::error\_codes::CONFIG

**Definition:** error\_codes.hpp:16


---

[ Stan Home Page ]

*© 2011–2014,
Stan Development Team.*
